# Prevalence and risk factors of workplace violence against health care workers in emergency department in Ismailia, Egypt

**DOI:** 10.11604/pamj.2017.26.21.10837

**Published:** 2017-01-17

**Authors:** Rasha Farouk Abdellah, Khaled Morsy Salama

**Affiliations:** 1 Department of Community, Environment and Occupational Medicine, Faculty of Medicine, Suez Canal University, Egypt; 2 Department of Emergency Medicine, Faculty of Medicine, Suez Canal University, Egypt

**Keywords:** WPV, emergency department, Egypt

## Abstract

**Introduction:**

Emergency department is one of the high-risk areas, where violence against health care workers (HCWs) is a prevalent and serious problem. Violence has negative effects on HCWs, and therefore on the quality of care provided in emergency department. This study aimed to determine the prevalence, types, sources and risk factors of violence reported by HCWs in emergency department.

**Methods:**

A cross-sectional study was conducted using a standardized questionnaire developed by the WHO. One hundred thirty four questionnaires were included in this study (94.4% response rate).

**Results:**

WPV was reported by 59.7% of HCWs. Verbal violence was the most reported (58.2%), compared to physical violence (15.7%). The most reported reasons for violence were waiting time and that patient and family expectations not being met. Only 29.5% of HCWs who experienced verbal violence and 23.8% of who experienced physical violence reported it to hospital authority. About 75% of HCW thought that work place violence could be prevented, and about 60% said that no action was taken against the attacker by hospital authority.

**Conclusion:**

Violence against HCWs in emergency department is a significant issue that cannot be ignored. There are multiple reasons. The key point in dealing with the problem is to treat its specific causes.

## Introduction

Work place violence (WPV) become a rising phenomenon especially among health care workers (HCWs) [1–4]. WPV was defined by the National Institute for Occupational Safety and Health (NIOSH) as: violent acts, including physical assaults, directed towards a person at work or on duty [5]. Verbal abuse through words, manner or tone, leaves the recipient feeling personally or professionally humiliated, attacked or devalued. Verbal abuse leaves no visible scars, but the emotional damage can be devastating [6]. Physical abuse is reported to occur within health care facilities four times more often than all other industries [4]. Among health care personnel, emergency workers are at a greater risk of violence than other hospital personnel, perhaps due to their frontline nature of works and to their 24-hour accessibility [1, 7, 8]. In addition to the high-stress environment, and lack of visible or trained security staff compared to other health care specialties [9]. Experiencing violence at work has many negative impacts both at the organizational and individual level, such as increase the perceptions of burnout, decreased job performance and job satisfaction, poor mental health, creating a hostile work climate and results in the suboptimal care to patients [2, 10]. The prevalence of WPV vary between countries. In Egypt, the prevalence of verbal abuse and physical abuse among nurses was 69.5% and 9.3% respectively [11]. In Saudi hospitals, more than two-thirds (67.4%) of HCWs reported they were victims of violence and nurses were more likely to be exposed to WPV than physicians (p<0.001) [12]. In Jordon, 75% of nursing staff in emergency departments some form of violence [2]. In Palestinian public hospitals, the majority of HCW (80.4%) reported exposure to violence in the previous 12 months; 20.8% physical and 59.6% non-physical [7]. In Turkish, 72.3% (141/195) of emergency staff had experienced some form of violence [13]. Although WPV touched high numbers of HCWs, the studies show that 80% of the affected didn’t report it [14]. Some reported reasons are fear of lack of support from the hospital authority or the absence of institutional reporting policies [15], the perception that violence is a part of the job and reporting will not benefit them [16]. Other beliefs that assaults may be viewed as worker negligence or poor job performance [14]. As WPV isn’t reported most of the time, workers are suffering in silence. Violence against HCWs is a varied and complex phenomenon. Some studies have reported factors relating to personal characteristics, such as being under the age of 30 [5, 7]. Other studies found that work-related factors, including: lack of communication, waiting time, lack of resources, medication error and lack of hospital policy against violence [3, 17] Triage area was the place where violence was most likely to occur, because it is the place of first encounter between the patient or family and HCWs [3, 18]. Different studies were conducted in many countries to find out the prevalence of WPV and identify the factors associated with it among HCW, but there is a lack of data to determine its magnitude among emergency department workers in Egypt. Therefore, the current study was aimed to determining it. Therefore, this work was conducted to identify (a) the prevalence of WPV among HCWs in emergency department, (b) the sources, (c) factors affecting violence experiences and the reporting of the incident.

## Methods

This cross-sectional study was conducted in emergency department in Suez Canal University hospital between April and May 2016. The hospital provides emergency services for 3 days per week. This tertiary care hospital provides free emergency care for the general population. The patients can go directly or referred from lower levels of health services. Data were collected through a standardized questionnaire developed by the World Health Organization [19]. The questionnaire was translated to Arabic (native language) and then back to English by different independent language experts to verify the consistency and content of translation. The questionnaire was divided into three sections. The first section had questions regarding personal and workplace characteristics as age, marital status, education, job category, duration of work and working hours. The second section had questions about verbal violence in the workplace in the last 12 months. The third section was related to physical violence. After explaining the aim of the study and obtaining consent, participants were asked to fill out the questionnaire. Participants were HCWs in emergency department for at least one year. They included various cadres of healthcare professionals directly involved in patient management such as physicians, nurses, secretaries and housekeepers workers. However, other health workers who have contact with patients but are not directly involved in patient management were excluded. These are record officers, security agents, cashiers, security agents and ward aides. Questionnaires were distributed to a convenience sample of 142 of HCWs employed in the emergency department. One hundred thirty four questionnaires were returned (94.4% response rate). Confidentiality and anonymity were maintained according to the regulations mandated by Research Ethics Committee of Faculty of Medicine Suez Canal University, in accordance with the Declaration of Helsinki. Data analysis was performed using SPSS 18.0 for windows. Descriptive statistics, including frequency distribution and percentages were made for most variables. Chi-Square test was used to examine the relationship between variables, Fisher exact test was used to examine the relationship between variables when the expected frequency is less than 5. P-values of less than 0.05 of the test measures were was considered statistically significant.

## Results

The socio-demographic characteristics of the study population are presented in Table 1. The total number of the studied population was 134 of HCWs. In the overall sample, there were 48.5% males, 50% of respondents are younger than 30 years. Nurses represent 50% of the study population and physicians represent 39.6%. Eighty (59.7%) of HCWs in emergency department were exposed to WPV during the past year. A total 78 (58.2%) were exposed to verbal violence: 24 (45.3%) physicians, 44 (65.7%) nurses and 10 (71.4%) of co-workers. Among those who reported verbal abuse, 60.9% were female, 37.8% were older than 35 years, 50.5% were married, and 67.6 % had an experience of 5 years or less in the health (Table 1). Among those who reported being physically attacked, 20% were males, 54.6% were younger than 30 years, 28.6% were co-workers, 13.4% nurses and 15.1% physicians, 28.6% were not married, 29.7% had experience of 5 years or less in emergency department (Table 1). The WPV characteristics were displayed in Table 2. Twenty-four percent reported that they experienced verbal aggression few times per month, 23.1% few times per week, and 15.4% once monthly. The patient relatives were responsible for 89.7% of verbal abuse and 90.5% of physical abuse. Regarding the work schedule, verbal abuse was more prevalent in evening shift (60.3%), while physical attacks were more prevalent in night shift (60.9%). As regard the reasons for violence, waiting time and patient and family expectations were the most reported. More than 70% of HCWs exposed to violence reported that violent attacks were preventable (Table 2).

**Table 1:** Demographic characteristics of the studied group exposed to WPV

Variables	Entire sample (n= 134)		Violence exposure (n= 80) (not mutually exclusive)			
			Verbal abuse (n=78 )		Physical attacks (n=21 )	
	No.	%	No.	%	No.	%
Job						
Physicians	53	39.6	24	45.3	8	15.1
Nurse	67	50.0	44	65.7	9	13.4
Coworkers	14	10.4	10	71.4	4	28.6
***X^2^***				6.18		2.03
***P***				0.046		0.36
Age (yrs)						
20 -	19	14.2	19	100	8	42.1
25 -	48	35.8	28	58.3	6	12.5
30 -	30	22.4	17	56.7	7	23.3
35 -	37	27.6	14	37.8	0	0.0
***X^2^***				19.98		18.62
***P***				0.00		0.000
Gender						
Male	65	48.5	36	55.4	13	20.0
Female	69	51.5	42	60.9	8	11.6
***X^2^***				0.41		1.78
***P***				0.52		0.18
Duration of work (yrs)						
< 5	37	27.6	25	67.6	11	29.7
5- 10	69	51.5	44	63.8	10	14.5
> 10	28	20.9	9	32.1	0	0.0
***X^2^***				10.02		10.81
***P***				0.007		0.004
Marital status						
Married	92	68.7	46	50.0	9	9.8
Not married	42	31.3	32	76.2	12	28.6
***X^2^***				8.13		7.70
***P***				.004		0.006
Smoking habits						
Non-smoker	102	76.1	60	58.8	13	12.7
Smoker	32	23.9	18	56.3	8	25.0
***X^2^***				0.06		2.76
***P***				0.79		0.09

# Coworkers include secretary and housekeepers workers

* Significant at p = 0.05

**Table 2:** Characteristics of WPV among the studied group

WPV characteristics	Verbal abuse (n=78 )		Physical attacks (n=21 )	
	N	%	N	%
**How often are you exposed to during the last year**				
Few times during last year	5	6.4	3	14.3
Once /month	12	15.4	7	33.3
Few times /month	19	24.4	6	28.6
Once /week	14	17.9	3	14.3
Few times /week	18	23.1	2	9.5
Every day	10	12.8	0	0.0
**The attacker was *(not mutually exclusive)***				
Patient	17	21.8	0	0.0
Patients’ relatives/ companion	70	89.7	19	90.5
Staff members/ Supervisors	20	25.6	5	23.8
**Most common time of WPV**				
8 am- 2 pm	13	16.7	0	0.0
2 pm- 8 pm	47	60.3	8	38.1
8 pm- 8 am	18	23.0	13	61.9
**Reasons for violence *(not mutually exclusive)***				
Waiting time	50	64.1	15	71.4
Overcrowding,	13	16.6	6	28.6
Patient and family expectations not being met	44	56.4	20	95.2
**Reporting of violence to hospital authority**				
Never	55	70.5	16	76.2
Sometimes	23	29.5	5	23.8
Always	0	0.0	0	0.0
**Could WPV have been prevented?**				
Yes	60	76.9	15	71.4
No	18	23.1	6	28.6
**Any action taken by hospital authority to investigate WPV cause**				
Yes	34	43.6	7	33.3
No	44	56.4	14	66.7

## Discussion

HCWs in emergency department experienced frequent and severe levels of WPV. In our study, eighty (59.7%) of HCWs in emergency department were exposed to WPV during the past year (Table 1). Our study findings are consistent with previous studies [2, 10, 12, 20]. The highly stressful situations and their direct contact with patients and their families were the main causes of verbal and physical abuse in emergency setting [21]. Among HCWs in emergency department, 65.7% of nurses, 45.3% of physicians and 71.4% of co-workers face verbal violence and aggression (Table 1). The highest rate of violence was toward co-workers, as they usually work at the entrance of emergency department where they make the first direct communication with patient and their relatives, this makes them vulnerable to violence incidents. Similarly, in Jordon, 55.5% of nurses were exposed to WPV [20]. In Nigeria, a study showed that 69.4% of health care professionals had experienced WPV. The results revealed that the highest prevalence was among the nurses (59.2%) followed by the doctors/dentists (57.4%) [10]. In Poland, 67% of nurses and 61% of physicians were exposed to aggressive behaviors. According to Poland study, the most common form of WPV was verbal aggression [22]. In United States, the prevalence of verbal violence against emergency department workers was reported as 75%, while physical violence reached 21% [23]. In the current study, males had higher percent of physical attacks compared to female nurses (20.0% vs 11.6%). WPV was higher in the age group below 25 year old. This is comparable to other studies, which reported that male nurses and less experienced nurses were likely to report both types of abuse physically and emotionally [7, 12]. Tolerant threshold vary from person to another, the higher prevalence in the younger age group can be attributed to lower threshold for insult and pain and less maturity compare to the older age group. In the current study, 60.3% of HCWs reported that verbal abuse is more in evening shifts while 61.9% reported that physical attacks were more in night shifts. The attacker person reported by HCWs in the current study was mainly the patient relatives about 90.0% followed by staff members and supervisors about 25.0% (Table 2). Our results are similar to those reported in the literature [1, 24, 25]. This indicated that when persons are exposed to critical health conditions or are in pain and wait for long times until seen by a physician or to receive medications, they and their relatives have high stress levels, feelings of anger and frustration which in turn manifested in the form of violence against others, as healthcare providers [12, 23]. Additionally, patients with psychosis and those under the influence of drugs or alcohol frequently present to the ED for treatment [16]. Some literature has been reported that medical staff may be responsible for emotional, verbal, and physical abuse against each other [26]. This is contrary to expectations, hospitals should be free from WPV and workers should work in a cooperative manner that provides a safe environment for both the patients and the co-workers themselves. As regard the reasons for violence, HCWs reported that waiting time and patient and family expectations were the main reasons. This finding may be because HCWs believe that the main factor that can decrease the violence and aggression in the emergency department is to improve resources. This finding is consistent with that reported by other studies [3]. In our study, about 75% of HCWs thought that violence attacks were preventable, while more than 70% never reported violence attacks to the hospital authority (Table 2). Many literatures declared that WPV was under-reported [24, 25]. This is a result of oppressed behavior, as nurses accept verbal abuse from all sources as part of the job. They do not believe that they have the power to prevent such events [27]. Also, being accustomed to workplace violence is the most stated reason for not reporting violence to the hospital administration or the authorities [26].

## Conclusion

Based on the results of our study, we conclude that WPV is a significant problem facing HCWs in emergency departments. Although most violence incidents were verbal, physical violence was not uncommon. These results therefore highlight the need to develop protocols for reporting, recognition, management and development of strategies to deal with WPV, and to carry out further control strategies for the problem. Further large-scale studies should be conducted to more closely examine the problem. Underestimation of magnitude of the problem may have occurred due to reluctance of HCWs to reports violence incidents and the possibility of recall bias. Lack of reporting is an important issue that needs to be addressed by hospital authority to document risks, plan interventions, and reduce such incidents. Educational program about WPV should be an essential part in the orientation programs for HCWs. This includes teaching HCWs about risk factors and situations that can lead to WPV, institution procedure for reporting WPV, communication skills and positive attitude.

### What is known about this topic

- Work place violence becomes a rising phenomenon especially among HCWs;
- Emergency workers are at a greater risk of violence than other hospital personnel;
- The prevalence of WPV vary between countries.

### What this study adds

- The prevalence of WPV among HCWs in emergency department, Egypt;
- The sources of WPV in emergency department;
- Factors affecting violence experiences and the reporting of the incident.
